# Power of Imagination

**Published:** 1872-10

**Authors:** P. H. Hayes


### POWER OF IMAGINATION.

P. H. HAYES, M. D.

A writer in the *Quarterly Journal of Psychological Medicine*, says, not many years ago a celebrated French novelist was writing a book, and issuing it in successive installments. At the same time a daughter of a Marquis was reading this story, wherein the heroine was described as passing into a decline. The fair reader, struck by the heroine's malady, began herself to grow thin, pale and hectic. Her father, alarmed at the effect upon his daughter, called upon the writer of novels, and besought him to restore the heroine of his story to health and happiness. He did as requested, and so, gradually as the heroine of the novel was brought up to new life and health, just so did the daughter of the Marquis recover health and spirits.

A woman of unusual intelligence used to send for me frequently at night, and when I reached her bedside would say, "Doctor, feel of my pulse ; put your hand here!" carrying my hand to the impulse of the heart. "Doctor, *is n't* my heart affected ? tell me now *truly* ;" and the strongest re-assurance I could give that it was not diseased, and the explanation of the real cause of its agitation would not last more than a day or two. Of all the organs which answers promptly to the feelings, the heart is most prompt—in it flesh and spirit closest blended. Fear weakens while it redoubles its movements. Rarely have these timid patients any heart disease at all; these palpitations are a mere symptom. Those who have real heart disease are, as a rule, strangely indifferent to it.

The old way was for the doctor to put his ear to the chest, hear a heart murmur, say "*heart disease*," and leave his patient in fear of death at any moment. This was most cruel ignorance, for of all the forms of real heart disease, there is but one of which this could be said, and with other forms a man may live a quarter of a century.

I once knew a young physician, who could increase his heart-beats almost to palpitation, by fixing his attention there. Per contra, a man's attention may be so powerfully withdrawn from self as not to feel pain, even when a powerful cause of pain is applied. Soldiers are sometimes not aware that they are wounded until the battle is over; and it is told of Campanella, that he could so forcibly abstract his attention from *fear* and from bodily pain, as to endure the rack itself with very little suffering.

Suppose a doctor should gravely tell his patient, "You have got a combination of diseases, sir; *embolism* of the liver, *congestion* of the *portal system* of veins, inflammation of *mucous membranes*, vital force all gone from the *organic nerves*, the *magnetic currents* are all reversed in your case, and there is a decided *regurgitation* on the left side of the heart." Would not this statement alone make him sick? I have talked with many chronic invalids, *word-struck*, sick in fact in no small degree, on doctor's *latin*, and this they betrayed by mixing in with their story the technical terms of the doctor, of the real meaning of which they knew no more than a parrot. Consciousness may tell us of pain, but it does not teach anatomy. A distinguished professor of medicine said one day to his class, "Paint the vein its whole course with iodine, the *name* of this disease is *Phlegmasia Alba Dolens*, a name not to be repeated in the hearing of your patient while she is weak!"

Some epidemic diseases are well known, and the name of these hold a fearful power over the imagination. Years ago a medical teacher told this story to his class: The *Good Genius* of the land of Egypt departed for a season from his country, and as he went, he met on the border of his dominion the *Plague*, and held this parley with him: Said the Good Genius, how many of my people are you going to destroy? Three thousand, said the Plague. It is well, answered the Good Genius, three thousand are all that I can spare. Time passed on; the Plague ended his work and departed, and as he went he met again the Good Genius on the border of his country, who reproached him for his broken faith; instead of three thousand you have taken of my people thirty thousand. Not so, said the Plague; I took but *three* thousand, and *Fear* took the rest.

Assalini, who treated the plague in Egypt in 1798-99, called it the *epidemic fever*, "to get rid of a name full of terror and often more mortal than the disease itself."

The reading of medical books, by sensitive people, which describe a disease they may dread, is a sure way of taking that disease by the imagination. These people are so apt to say—"There, that's my case exactly." So said Assalini to his physicians even, who treated the plague with him in Egypt, if you are fond of reading, choose amusing books, but not those that treat of the plague.

No passion is more contagious than fear. So we read in Deuteronomy, 20th, 8th, that when Israel went to war, the officers were commanded to separate the "fearful and faint-hearted; let him go and return unto his own house, lest his brethren's heart faint as well as his heart."—Hence, also, the bad effects from invalids talking over their cases together, and if already fear-stricken, as to themselves, talk they will. It is the all-absorbing theme with them, and they ask the doctor over and over again, if he *ever* saw such a case as theirs before. Now, such is the contagion of fear, and such is the power of sympathy, that invalids who talk much with invalids, are apt to get their cases mixed. So, when I saw Mr. E., who has dyspepsia, very earnestly talking with D., who has *sciatica*, I was more amused than surprised, when next time I met E., he asked me very seriously, if I did not think there was some *sciatica* about his case.

In my lifetime I have met many invalids slowly and insensibly perishing for the want of a better nutrition, because, following some contemptible theory, they were afraid to eat anything but coarse, weak preparations of food. Some of these monomaniacs wanted to weigh this food, so they might not get the fraction of an ounce more than the theory called for, and some would eat of it only twice a day. Their stomachs grew weak from this course, and this very weakness they thought required a still *milder diet!* Of all the classes of fear-stricken people, none have I ever seen more "stiff set" in their delusion.—

Slowly down they go, and die from nothing else than this sort of painless decay of all the powers and actions of life.

Thousands of invalid people read the quack advertisements which find their way into every household. Many of these advertisements do incalculable harm, by directing a sufferer's attention to the study of his own case through his fears, and inciting him to turn tinker to his own constitution.

There are men calling themselves doctors, in almost every community, who understand to a point wherein their power over the sick lies.—They know perfectly well how to hold an invalid in suspense by the power of fear. They know perfectly well that their power over the sick does not lie in their ability to cure them, but in a mild degree of terrorism. These doctors are able to play a thousand variations on this *E* fibre of human weakness, and then retire to say, how people love to be dosed and humbugged!

In these illustrations of morbid imagination, fear gives the stroke and causes the suffering. How far fear is voluntary and how far involuntary, it may not be easy to determine; but it would seem that if instinctive fear can be almost wholly overcome by discipline, there would be no room for doubt as to those which are imaginary.

Savages bear bodily torture apparently without fear or the slightest flinching, and I have heard of a juggler in India, who, through long discipline, could bear to be pinched, bruised, cut or burned, and not flinch or quiver.

Now don't lose the moral, while I tell you that a sharp Yankee brought down this same juggler, by putting a bottle of hartshorn to his nose. Why did the *ammonia* bring down the juggler, and spoil as it did a reputation of eighteen years' growth? Simply because he had not hardened the nerves of his nose. Is there not some way of hardening our moral constitution against all the protean forms of fear, which consciously or unconsciously, tyrannize over the imagination, double our sufferings, nay, constitute all the suffering in hosts of cases.

